# Supplementary material for: Hox dosage contributes to flight appendage morphology in Drosophila
Source: Nat Commun. 2021 May 17;12:2892. doi: 10.1038/s41467-021-23293-8 (PMC8129201; doi:10.1038/s41467-021-23293-8)
Supplement: Supplementary file 1 — Supplementary Information [file 41467_2021_23293_MOESM1_ESM.pdf]

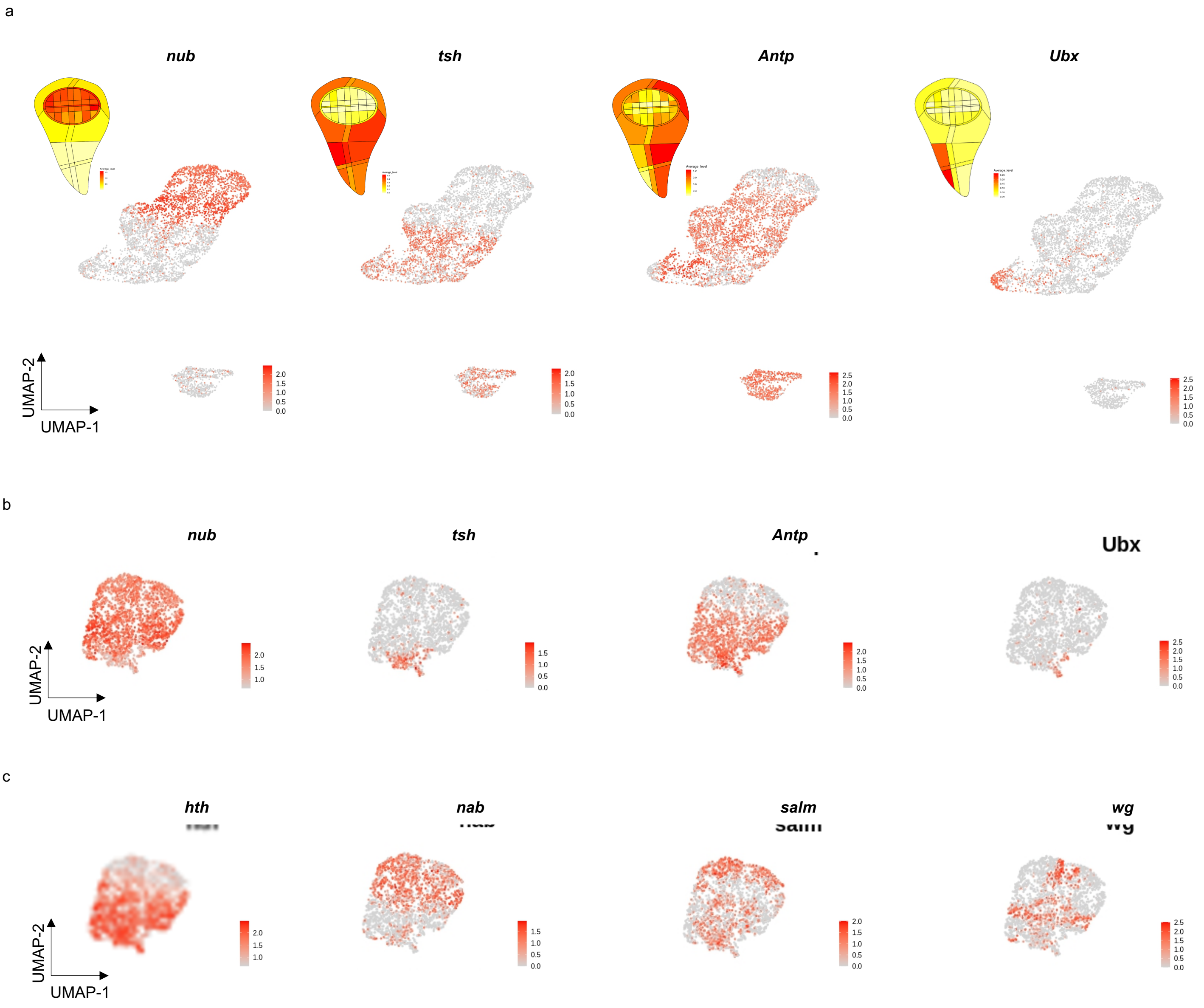

Supplementary Figure 1. Wild-type L3 wing disc single-cell RNA-seq data recapitulate the ANTP expression profile obtained from immunostaining. a. Virtual discs built to predict gene expression pattern of *nubbin* (*nub*), *teashirt (tsh), Antp,* and *Ultrabithorax* (*Ubx*). The yellow-to-dark red colour code symbolises the weak-to-high gradient expression level. The corresponding expression level deduced from RNA-seq in the group of wing epithelial cells is shown (with a light-to-dark red gradient symbolizing low-to-high expression levels). The normalized expression level is plotted on a UMAP projection from the whole wing disc. A small group of myoblast cells is also shown below for comparison with the group of wing epithelial cells. **b.** UMAP projections of pouch cells sub-set by marker gene *nub* and colored by the expression levels of *nub*, *tsh, Antp* and *Ubx.* **c.** UMAP projections of pouch cells coloured by the expression levels of *homothorax* (*hth*), *nab*, *spalt-major* (*salm*), and *wingless* (*wg*).

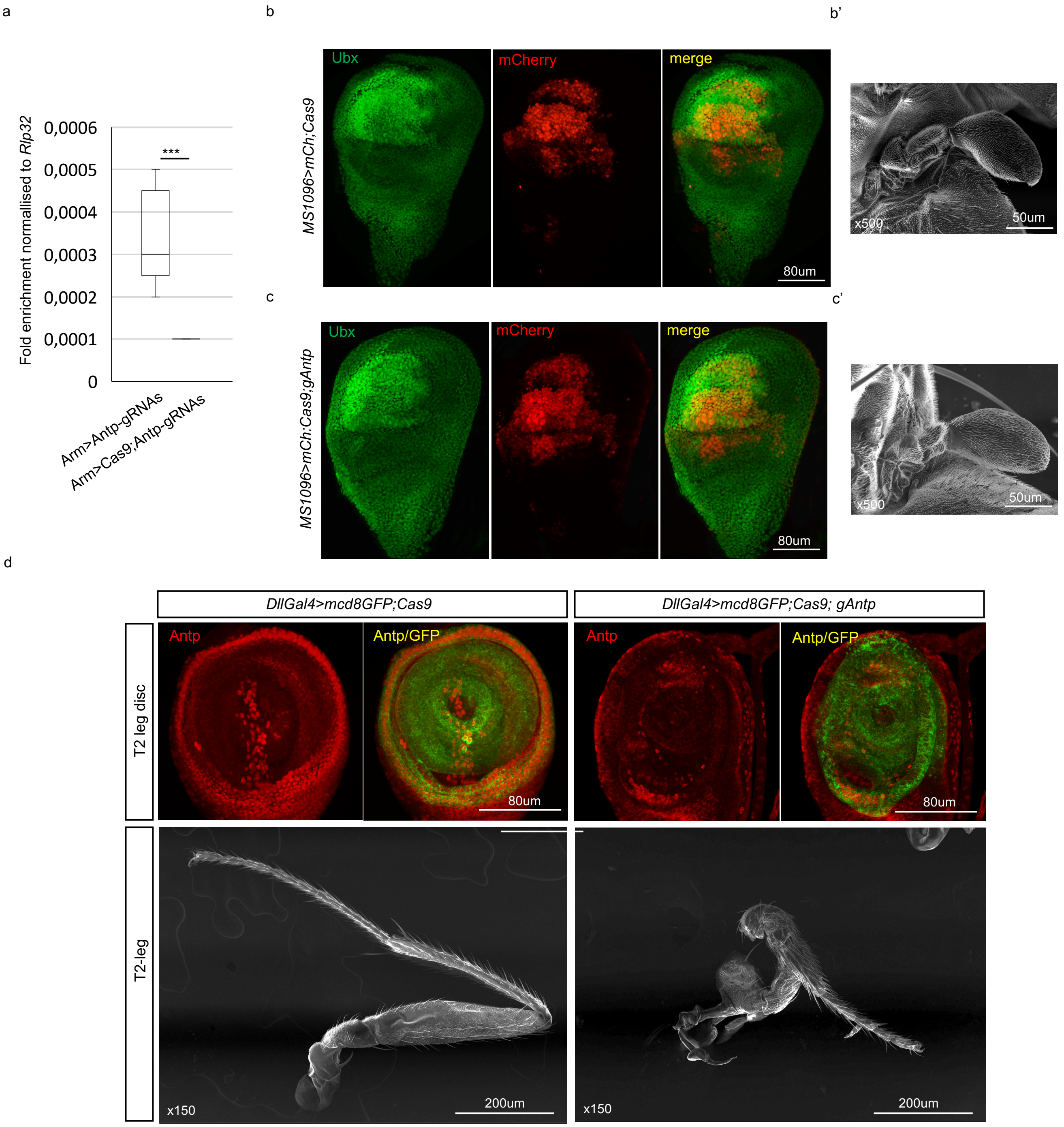

Supplementary Figure 2. Specificity of the *gRNAs* against *Antennapedia* (*Antp*). a. Boxplot representation of qPCR against *Antp* upon expression of *Antp-gRNAs* and *Cas9* with the ubiquitous *armadillo(arm)-Gal4* driver in the embryo. Compared to the controls (ns, non-significant), *Antp* expression is specifically affected (75% loss on average) when both *Antp-gRNAs* and *Cas9* are co-expressed (unpaired two-tailed t-test ****P*value=3.44e-06). Boxplots indicate 25th and 75th percentiles, whiskers show ±1.5*IQR and center line depicts median of 3 biological replicates. W: white minus background. **b.** Endogenous Ultrabithorax (Ubx) expression (green) is not affected in *MS1096*-driven mCherry (mCh, red) and *Cas9* in the haltere disc (n=10 from two independent experiments). **b'**. Corresponding illustrative SEM acquisition of a non-affected adult haltere (n=15 from two independent experiments). **c.** Endogenous Ubx (green) is not affected upon the co-expression of *mCherry*, *Antp-gRNA* and *Cas9* with the *MS1096-Gal4* driver (n=10 from two independent experiments). **c'.** No phenotype is observed in the adult haltere (n=15 from two independent experiments). **d.** Specificity of the *Antp-gRNAs* in the leg disc. The *Distalless* (*Dll*)*-Gal4* expressing cells are traced with the mcd8-GFP (green) reporter in the T2 leg disc that also expresses Antp (red). Antp is lost upon co-expression of *Antp-gRNAs* and *Cas9* (n=7 from two independent experiments), which led to adult leg phenotypes that are reminiscent of classical *Antp*-mutant phenotypes (n=15 from two independent experiments). All illustrative confocal and SEM images were systematically reproduced from the different experiments.

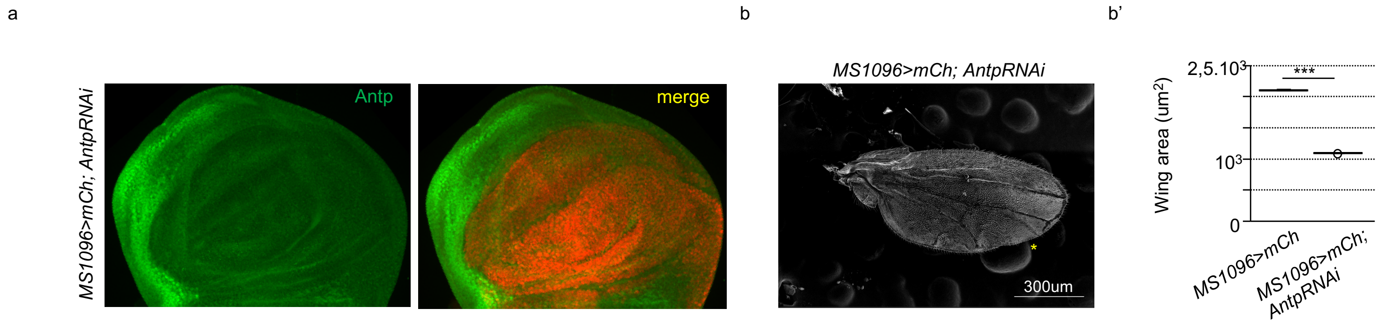

Supplementary Figure 3. RNAi against *Antp* reproduces similar wing phenotypes as with Crispr/cas9. a. Expression of a RNAi against *Antp* with the *MS1096-Gal4* driver (red) induces a loss of Antp (green) expression in the wing disc pouch (n=10 from two independent experiments). **b.** Illustrative SEM acquisition of the adult wing of an individual expressing *Antp-RNAi* with *MS1096*. These individuals display wing margin phenotypes (yellow star) and wing size reduction, as previously observed when using Crispr/Cas9 (n=17). Illustrative confocal and SEM images were systematically reproduced from the different experiments. **b'.** Boxplot representation of the quantification of the wing area in *Antp-RNAi* individuals and showing a significant reduction of the wing size when compared to control individuals (unpaired two-tailed t-test ****p*value=7.35e-20). Boxplots indicate 25th and 75th percentiles, whiskers show ±1.5*IQR and center line depicts median of 3 biological replicates.

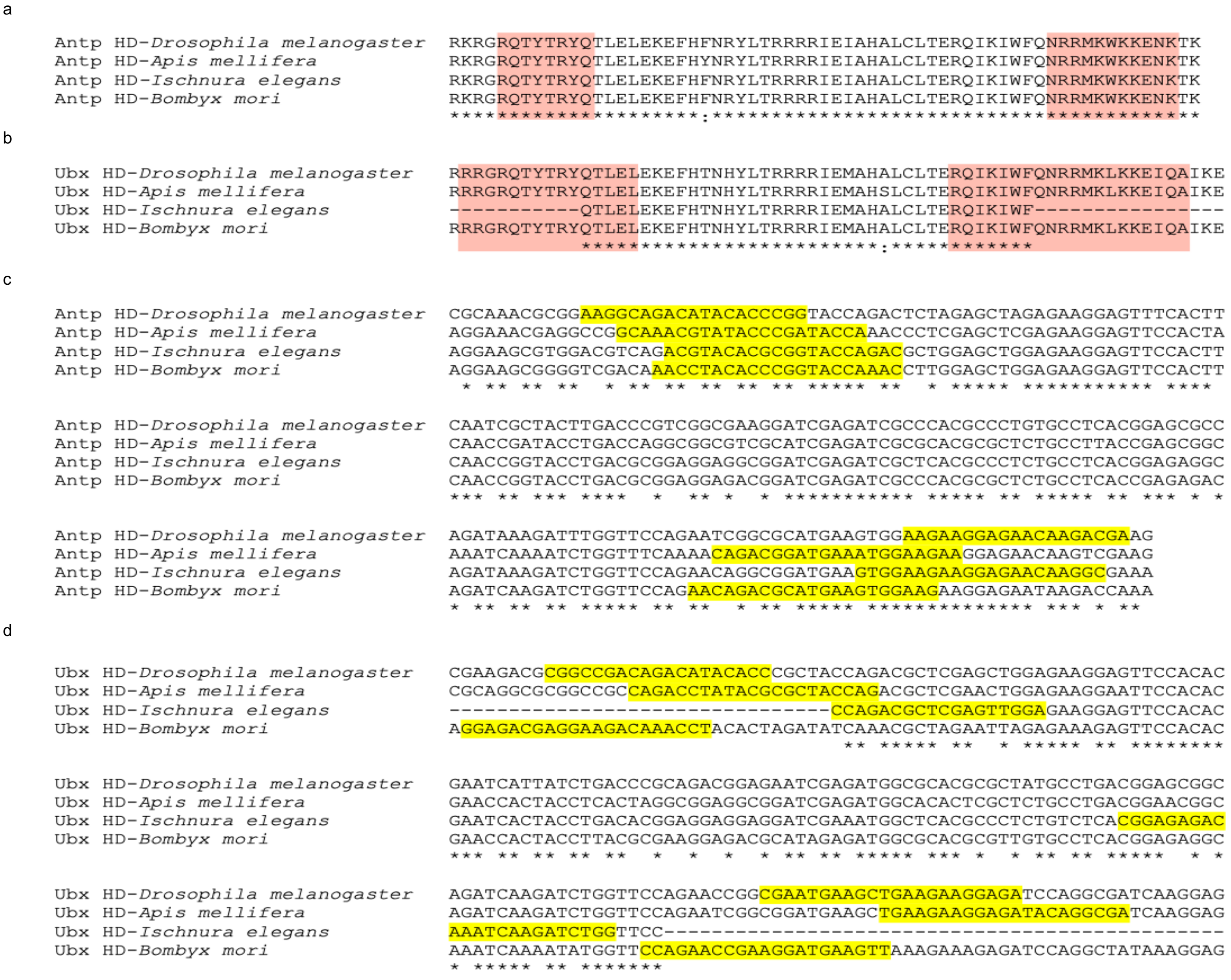

Supplementary Figure 4. Design of qPCR primers. a-b. Amino-acid sequences alignments for Antp (A) and Ubx (B) homeodomains (HD) in *Drosophila melanogaster*, *Apis mellifera*, *Ischnura elegans* and *Bombyx mori*. Red boxes frame the amino-acid sequences in which all primers were chosen except for Ubx in *I. elegans*. **c-d.** Nucleotide sequence alignment of Antp (C) and Ubx (D) HDs from different insect species, as indicated. Primers used for qPCR are underlined in yellow.

a b c

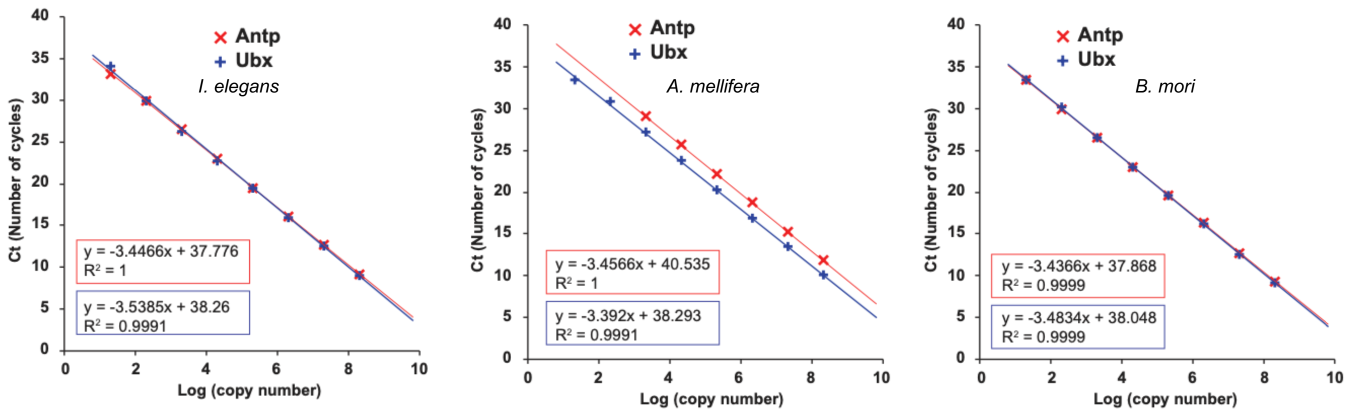

Supplementary Figure 5. qPCR primers analysis. Standard affinity curves for *Antp* (in red) and *Ubx* (in blue) in *Apis mellifera* (a), *Bombyx mori* (b), and *Ischnura elegans* (c). Line graphs show the cycle threshold (Ct) according to the log of each initial copy number. Equations and R^2 for linear regression are indicated.

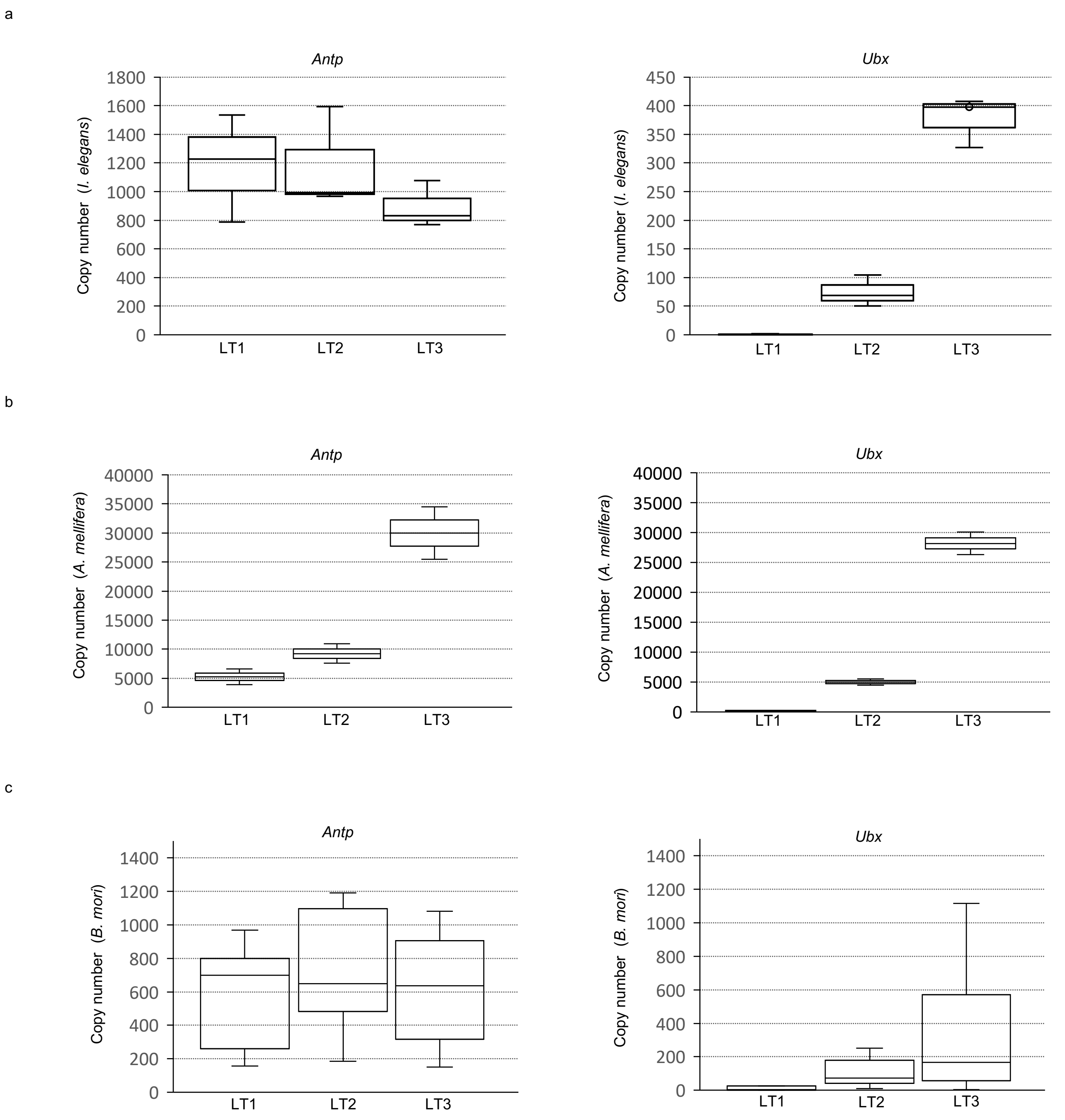

Supplementary Figure 6. Boxplot representation of control RT-qPCRs of *Antp* and *Ubx* in the leg primordia of the first (LT1), second (LT2) and third (LT3) thoracic segment in *Ischnura elegans* (a), *Apis mellifera* (b) and *Bombyx mori* (c). Boxplots indicate 25th and 75th percentiles, whiskers show ±1.5*IQR and center line depicts median of 3 biological replicates.
