# Supplementary material for: Hox dosage contributes to flight appendage morphology in Drosophila
Source: Nat Commun. 2021 May 17;12:2892. doi: 10.1038/s41467-021-23293-8 (PMC8129201; doi:10.1038/s41467-021-23293-8)
Supplement: Supplementary file 2 — Reporting Summary [file 41467_2021_23293_MOESM2_ESM.pdf]

nature research

Corresponding author(s): Merabet

Last updated by author(s): Apr 10, 2021

Reporting Summary

Nature Research wishes to improve the reproducibility of the work that we publish. This form provides structure for consistency and transparency in reporting. For further information on Nature Research policies, see our Editorial Policies and the Editorial Policy Checklist.

Statistics

For all statistical analyses, confirm that the following items are present in the figure legend, table legend, main text, or Methods section.

n/a	Confirmed	
☐	☒	The exact sample size (*n*) for each experimental group/condition, given as a discrete number and unit of measurement
☐	☒	A statement on whether measurements were taken from distinct samples or whether the same sample was measured repeatedly
☐	☒	The statistical test(s) used AND whether they are one- or two-sided<br>*Only common tests should be described solely by name; describe more complex techniques in the Methods section.*
☒	☐	A description of all covariates tested
☒	☐	A description of any assumptions or corrections, such as tests of normality and adjustment for multiple comparisons
☐	☒	A full description of the statistical parameters including central tendency (e.g. means) or other basic estimates (e.g. regression coefficient) AND variation (e.g. standard deviation) or associated estimates of uncertainty (e.g. confidence intervals)
☐	☒	For null hypothesis testing, the test statistic (e.g. *F*, *t*, *r*) with confidence intervals, effect sizes, degrees of freedom and *P* value noted<br>*Give P values as exact values whenever suitable.*
☒	☐	For Bayesian analysis, information on the choice of priors and Markov chain Monte Carlo settings
☒	☐	For hierarchical and complex designs, identification of the appropriate level for tests and full reporting of outcomes
☒	☐	Estimates of effect sizes (e.g. Cohen's *d*, Pearson's *r*), indicating how they were calculated

Our web collection on statistics for biologists contains articles on many of the points above.

Software and code

Policy information about availability of computer code

Data collection	No software was used for data collection
Data analysis	Fiji-2, CrossMap, Seurat R package v3.1, UMAP

For manuscripts utilizing custom algorithms or software that are central to the research but not yet described in published literature, software must be made available to editors and reviewers. We strongly encourage code deposition in a community repository (e.g. GitHub). See the Nature Research guidelines for submitting code & software for further information.

Data

Policy information about availability of data

All manuscripts must include a data availability statement. This statement should provide the following information, where applicable:

- Accession codes, unique identifiers, or web links for publicly available datasets
- A list of figures that have associated raw data
- A description of any restrictions on data availability

The datasets generated during and/or analysed during the current study are available from the corresponding author on reasonable request. Source data are provided for the Figures 2, 5, 6, 7 and Supplementary Figures 2, 3 and 6.

Field-specific reporting

Life sciences study design

All studies must disclose on these points even when the disclosure is negative.

Sample size	A minimum of 10 specimen was used for each experiment, making a total of 20 imaginal discs per sample, with a minimum of two replicates for each condition. This minimum number of specimen takes into account the accessibility of the biological material (based on micro-dissection) and the requirement of having statistical enrichment and biological significance.
Data exclusions	No data were excluded.
Replication	All statistical analyses were performed from at least two different bological replicates. All attemps at replication were successfull.
Randomization	Biological samples were randomly used and biological replicates were from different populations. Experiments were randomly repeted by different students.
Blinding	Investigators were not systematically blinded. In particular, blinding was apply to all data related to the Figures 1-6, but not to the data related to the Figure 7 (immunostaining and RT-qPCR in non-Drosophila species). It was considered as not critical given that the results were statistically highly significant.

Reporting for specific materials, systems and methods

We require information from authors about some types of materials, experimental systems and methods used in many studies. Here, indicate whether each material, system or method listed is relevant to your study. If you are not sure if a list item applies to your research, read the appropriate section before selecting a response.

Materials & experimental systems

n/a	Involved in the study	
☐	☒	Antibodies
☒	☐	Eukaryotic cell lines
☒	☐	Palaeontology and archaeology
☐	☒	Animals and other organisms
☒	☐	Human research participants
☒	☐	Clinical data
☒	☐	Dual use research of concern

Methods

n/a	Involved in the study	
☒	☐	ChIP-seq
☒	☐	Flow cytometry
☒	☐	MRI-based neuroimaging

Antibodies

Antibodies used

Primary antibodies used were:
- anti-Antp 4C3, DSHB, reference AB 528082
- anti- Antp 8C11, DSHB, reference AB 528083
- anti-Ubx FP3.38, DSHB, reference FP3.38
- anti-Ubx/ABD-A, DSHB, reference FP6.87
- anti-Wg 4D4, DSHB, reference AB 528512
- anti-Spalt, generously provided by the laboratory of Jonathan Enriquez (IGFL, Lyon, France)
- anti-Nubbn, generously provided by the laboratory of Jonathan Enriquez (IGFL, Lyon, France)
- anti-Hth, generously provided by the laboratory of Jonathan Enriquez (IGFL, Lyon, France)
- anti-GFP, Chromotek, reference PABG1

Secondary antibodies used were:
- anti-rabbit IgG Alexa fluor 488, ThermoFischer, reference A-11008
- anti-mouse IgG Alexa fluor 488, ThermoFischer, reference A-11001
- anti-rabbit IgG Cyanine3, ThermoFischer, reference A10520
- anti-mouse IgG Cyanine3, ThermoFischer, reference A10521

Validation

- anti-Antp 4C3, statement from DSHB, validated for Gel Supershift, Immunofluorescence, Immunohistochemistry
- anti-Antp 8c11, statement from DSHB, validated for Immunofluorescence, Immunohistochemistry

anti-Ubx FP3.38, statement from DSHB, validated for Chromatin Immunoprecipitation, Immunofluorescence, Immunohistochemistry, Immunoprecipitation, Western Blot
- anti-Ubx/ABD-A, statement from DSHB, validated for Immunofluorescence, Immunohistochemistry, Immunoprecipitation, Western Blot
- anti-Wg 4D4, statement from DSHB, validated for Immunofluorescence, Immunohistochemistry, Immunoprecipitation, Western Blot
- anti-Spalt, validated by Immunofluorescence in Rosa Barrio, Jose F. de Celis, Slava Bolshakov, Fotis C. Kafatos. Identification of

Regulatory Regions Driving theExpression of theDrosophila spaltComplexat Different Developmental Stages. Developmental Biology 215, 33-47 (1999).
- anti-nubbin, validated by Immunofluorescence in M Ng, F J Diaz-Benjumea, J P Vincent, J Wu, S M Cohen. Specification of the wing by localized expression of wingless protein. Nature May 23;381(6580):316-8. 1996.
- anti-Hth, validated by Immunofluorescence in Casares F, Mann RS. Control of antennal versus leg development in Drosophila. Nature. Apr 16; 392(6677):723-6. 1998.
- anti-GFP, statement from Cnromotek, validated for Immunofluorescence, Immunohistochemistry

Animals and other organisms

Policy information about studies involving animals; ARRIVE guidelines recommended for reporting animal research

Laboratory animals	yelow white fruit flies, males or females, 5 days old
Wild animals	No wild animals were used in the study
Field-collected samples	no field collected samples were used in the study
Ethics oversight	no ethical approvance was required (not necessary for fruit flies)

Note that full information on the approval of the study protocol must also be provided in the manuscript.
